# *In vitro* differentiation of human pluripotent stem cells into the B lineage using OP9-MS5 co-culture

**DOI:** 10.1016/j.xpro.2021.100420

**Published:** 2021-03-31

**Authors:** Simon E. Richardson, Roshanak Ghazanfari, Jyoti Chhetri, Tariq Enver, Charlotta Böiers

**Affiliations:** 1 Wellcome-MRC Cambridge Stem Cell Institute, University of Cambridge, Cambridge CB2 0AW, UK; 2 Department of Haematology, University of Cambridge, Jeffrey Cheah Biomedical Centre, Cambridge CB2 0AW, UK; 3 UCL Cancer Institute, 72 Huntley Street, London WC1E 6DD, UK; 4 Division of Molecular Hematology, Department of Laboratory Medicine, Lund Stem Cell Center, Lund University, 221 84 Lund, Sweden; 5 Division of Molecular Medicine and Gene Therapy, Department of Laboratory Medicine, Lund University, 221 84 Lund, Sweden

**Keywords:** Cell culture, Stem Cells, Cell Differentiation

## Abstract

*In vitro* differentiation of human pluripotent stem cells (hPSCs) offers a genetically tractable system to examine the physiology and pathology of human tissue development and differentiation. We have used this approach to model the earliest stages of human B lineage development and characterize potential target cells for the *in utero* initiation of childhood B acute lymphoblastic leukemia. Herein, we detail critical aspects of the protocol including reagent validation, controls, and examples of surface markers used for analysis and cell sorting.

For complete details on the use and execution of this protocol, please refer to Boiers et al. (2018).

## Highlights

• Protocol to derive pro- and pre-B cells from human pluripotent stem cells
• Step-by-step guide to growing and validating hPSCs and OP9 & MS5 stroma
• Analysis of hematopoietic and B lineage progenitors by flow cytometry

## Graphical Abstract

**Figure:**
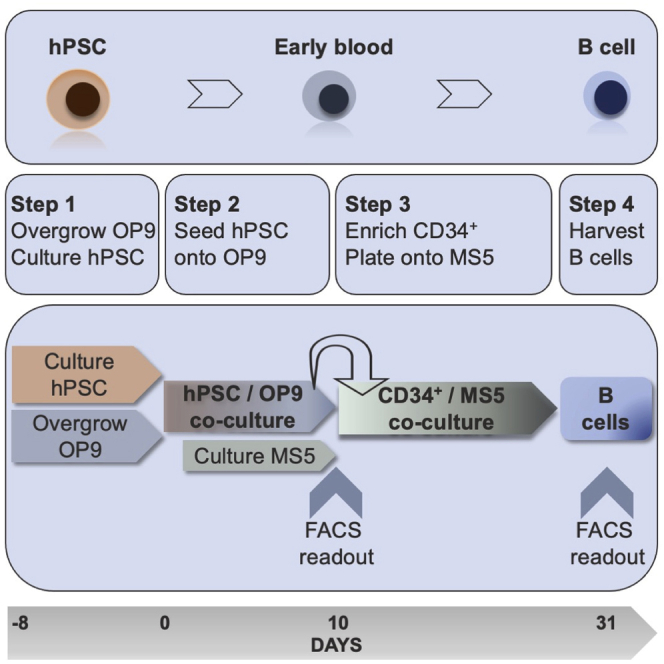

## Before you begin

### Establish feeder-free hPSC culture

**Timing: 1–2 weeks**

1. Identify a suitable hPSC line.

***Note:*** It is widely considered that different hPSC lines can exhibit lineage biases upon differentiation, which raises the possibility that certain cell lines will exhibit limited or no B lineage differentiation potential. When establishing this protocol, we therefore strongly advise validating the system using a well-maintained hPSC line with known B lineage potential. We have demonstrated robust B lineage output from the widely available H1 human embryonic stem (ES) cells and ShIPS-MIFF3 (and to a lesser extent MIFF1) human induced pluripotent stem cell (iPSC) lines.

2. Prepare Matrigel-coated 6 well plates.
  a. Thaw stock vial of Matrigel on ice at 4°C for 8**–**12 h.
  b. Aliquot working stocks into chilled 15 mL conical tubes, using chilled tips on ice according to the dilution factor supplied by the manufacturer and store at −20°C.
  c. Thaw Matrigel aliquots on ice and resuspend in an appropriate volume of chilled DMEM/F12 basal media using a chilled serological pipette. Dispense 1 mL of 1**×** Matrigel per well of a 6 well plate to coat the base. These plates can be stored at 4°C for up to 7 days and used after 1 h of warming at 37°C.
  d. Remove Matrigel immediately prior to use; do not allow plates to desiccate.

**CRITICAL:** Matrigel must be handled at ice cold temperatures until applied to the surface to be coated. See manufacturer’s instructions for more details.

3. Prepare StemFit (Basic04) hPSC media
  a. Thaw the media at 4°C for 8**–**12 h.
  b. Aliquot in 50 mL or 15 mL conical tubes and store at −20°C.
  c. Thaw before use and add 10 ng/mL human basic Fibroblast Growth Factor (hbFGF).

***Note:*** Avoid prolonged incubation at 37°C as this degrades hbFGF. StemFit media is not used beyond 7 days of storage at 4°C. Higher concentrations of hbFGF may be needed.

***Optional:*** 1% Penicillin/Streptomycin can be added to the media.

***Alternative:*** We have also successfully used hPSCs grown in mTeSR1/mTeSR Plus media in this protocol, used according to manufacturer’s instructions.

4. Prepare 1000**×** solution of Y27632 (ROCK inhibitor).
  a. Reconstitute Y27632 powder in distilled water to a final concentration of 10 mM, aliquot into sterile tubes and store at −20°C.
  b. Add 1:1000 of stock Y27632 to StemFit media to create a final concentration of 10μM. Working aliquots can be stored at 4°C for up to 72 h and should not be freeze-thawed.

***Note:*** Y27632 (ROCK inhibitor) containing media should be replaced with fresh basal media after 24 h as it affects hPSC colony morphology, however, we sometimes extend to 48h where hPSC viability is particularly compromised (e.g., difficult thawing or single cell cloning).

***Note:*** hPSC lines tolerate single cell dissociation poorly. We therefore recommend adding Y27632 to the hPSC media during thawing, routine passage, and single cell manipulations (e.g., flow cytometry, cloning by limiting dilution etc.).

5. Thaw hPSC line(s).
  a. Prepare an appropriate aliquot of Y27632-containing StemFit media as above.
  b. Rapidly thaw a cryovial of hPSCs in a 37°C water bath. Transfer cells to a 15 mL conical tube, ensuring minimal break up of cell clumps.
  c. Slowly dilute freezing media with the addition of 10**×** excess volume of fresh, warm StemFit.
  d. Centrifuge for 5 min at 200 g.
  e. Remove Matrigel from 6 well plate and replace with 1.5 mL Y27632-containing StemFit.
  f. Remove the supernatant and gently resuspend the cell pellet in 1 mL Y27632-containing StemFit. Transfer to a well of a 6 well plate in a final volume of 2.5 mL StemFit.
  g. Feed daily with fresh media, avoiding exposure to more than 24 h (max 48 h if concerns regarding viability) Y27632.

***Note:*** Cells grown in StemFit Basic04 media have regular complete media changes, but do not require daily feeding. See manufacturer’s instructions for recommended passaging/feeding schedules (https://www.nippongenetics.eu/en/product/stemfit-basic04/).

***Note:*** We routinely freeze hPSCs in 90% Knock Out Serum Replacement (KOSR)/10% DMSO.

**Figure 1:**
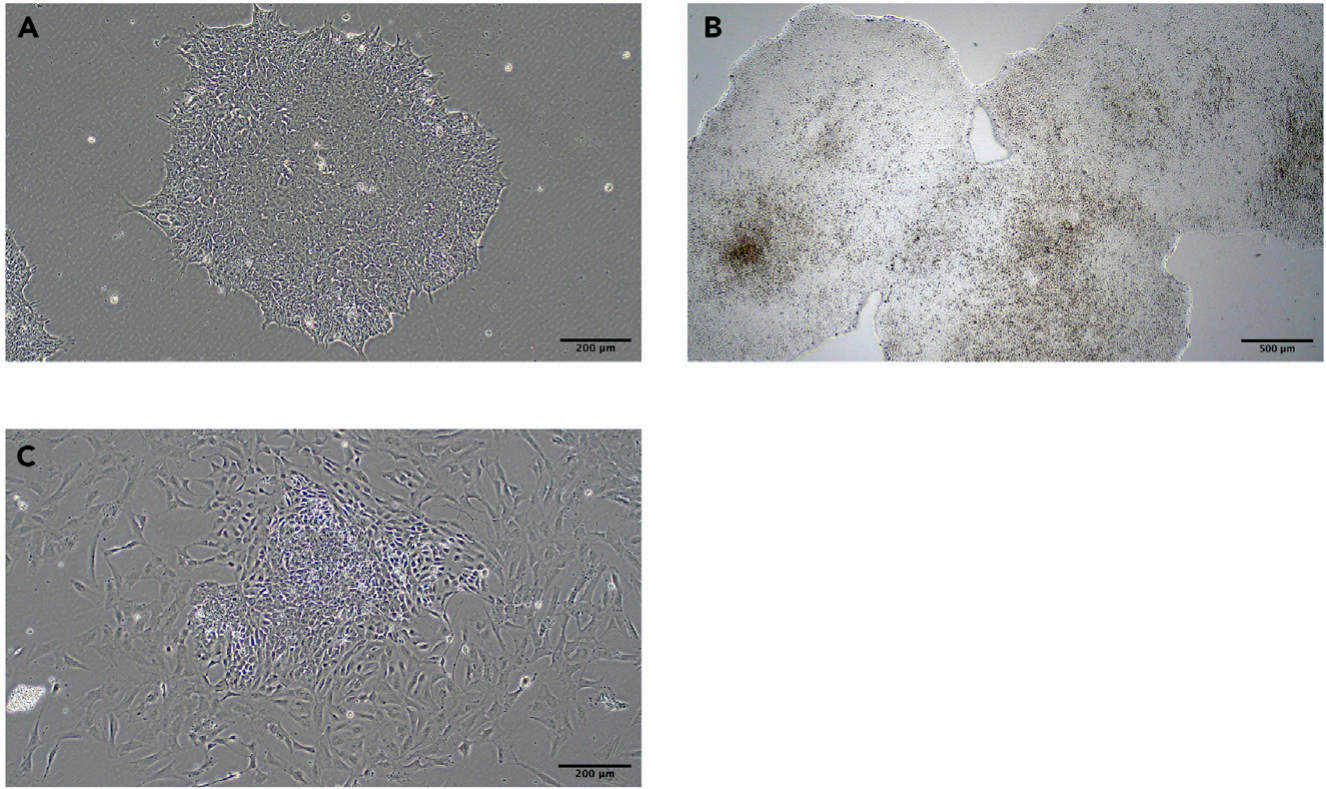
Morphology of hPSCs (A) Photo of an undifferentiated MIFF3 IPS clone on Matrigel and in StemFit media. (B) Photo of an undifferentiated MIFF3 IPS clone on Matrigel and in StemFit media just before passage. (C) Differentiated MIFF3 hIPS colony unsuitable for use in differentiation. Microscope Olympus, CKX53. Scale bars were added using ImageJ software.
  b. Remove StemFit media and replace with 1 mL gentle cell dissociation reagent (GCDR).
  c. Incubate at 37°C for 5min.
    ***Note:*** The time required in GCDR varies depending on the hPSC line used.
  d. Gently remove GCDR avoiding disturbing the colonies and replace with 1 mL Y27632-containing StemFit media.
  e. Mechanically scrape hPSC colonies using a cell scraper into clumps. If there are large visible clumps, gently titurate the cell suspension with a 1 mL pipette tip and transfer to a new Matrigel-coated 6 well plate at an appropriate cell density.
    ***Alternative:*** For passaging hPSCs, GCDR can be replaced with ReLeSr, which preferentially detaches undifferentiated hPSC cells.
    ***Note:*** Centrifugation during passage is not necessary and cells can be split directly into a new plate. The precise seeding density depends on the characteristics of the hPSC line being used; in our hands a 1:4 to 1:10 split would be routine for H1 hES or MIFF3 hIPS cells.
    ***Note:*** In our experience hPSCs are not sufficiently established for B cell differentiation for at least 2 passages (about 10 days) after thawing.

6. Routine passage of hPSCs.
  a. hPSCs are passaged when colonies are large or showing signs of differentiation. In general, well-maintained colonies that are fed regularly and passaged in a timely way do not require manual or chemical removal of differentiated cells (Figures 1A–1C).

### Maintenance and storage of differentiation-competent OP9 and MS5 stroma

**Timing: 2 weeks**

**Figure 2:**
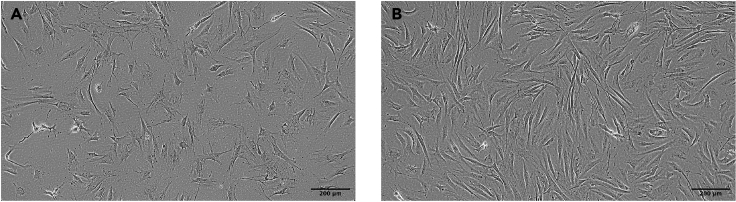
Morphology of OP9 stroma in maintenance phase (A) Sparse OP9 cells in maintenance. (B) OP9 prior to passage at 80% confluency. Microscope Olympus, CKX53. Scale bars were added using ImageJ software.

**CRITICAL:** The quality of OP9 stroma used is one of the most important aspects of this protocol. OP9 during maintenance should not be allowed to overgrow and are split approximately every 4 days to maintain a morphological profile shown in Figure 2A and 2B. Failure to maintain good quality OP9 will result in morphological changes such as increased adiposity and an associated reduction or failure to generate hematopoietic progenitors.

**CRITICAL:** The choice of plastic used in the tissue culture plate can affect differentiation (see Key Resources Table).

7. Reserve batch of qualified FBS.

**CRITICAL:** It is important to use qualified FBS and different batches can affect both the morphology of OP9 and the yield of B cells. We therefore recommend reserving batches of qualified FBS and testing for adequate B lineage output prior to committing to purchase.

***Note:*** FBS used in this protocol does not require heat inactivation.

8. Make OP9 maintenance (OP9-M) media as per instructions below.

**CRITICAL:** We have found that the use of αMEM made from powder is essential.

**Figure 3:**
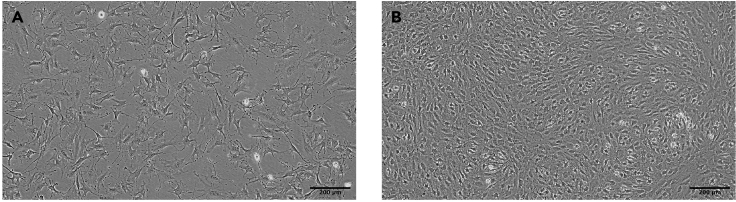
Morphology of MS5 stroma in maintenance phase (A) MS5 stromal morphology prior to passage. (B) MS5 stromal morphology when overgrown; do not use in further experiments. Microscope Olympus, CKX53. Scale bars were added using ImageJ software.
  f. On passaging, carefully remove media, wash with 5 mL DPBS.
  g. Add 5 mL trypsin (0.05% in DPBS, 5 mM EDTA) and incubate at 37°C for 5 min.
  h. Quench trypsin with 5 mL MS5-M media and titurate until cells have visibly detached.
  i. Transfer pellet to 15 mL conical tube, centrifuge at 300 g for 5min.
  j. Split pellet 1:4**–**1:12 into a new gelatinized 75 cm^2^ flasks.
    ***Note:*** Once good quality cultures are established freeze aliquots of low passage cells in 90% qualified-FBS/10% DMSO.

9. Expand stocks of OP9 stroma.
  a. Pre-gelatinize 10 cm tissue culture dish with 5 mL 0.1% bovine gelatin for at least 12 h at 4°C.
    **CRITICAL:** The use of pre-gelatinized plates is essential to maintain good stromal morphology and integrity during differentiation. We routinely gelatinize plates for 1**–**4 days at 4°C prior to passage.
  b. Thaw cryovial of OP9 in 10**×** volume of OP9-M media, transfer to 15 mL conical tube and centrifuge at 300 g for 5min.
  c. Completely remove gelatin from 10 cm plate and seed OP9 cell pellet in 10 mL OP9-M media.
  d. Monitor daily for growth and passage to maintain morphology as per Figures 2A and 2B. Once established routine passage is typically a 1:4 split every 4 days.
    ***Note:*** Early passage OP9 may grow faster, requiring passaging more frequently than 4 days. Do not allow OP9 maintenance cultures to overgrow.
  e. When passaging, remove and discard all media, wash twice gently with 5 mL DPBS to remove all remaining media (this will help to maximize the effect of trypsin).
  f. Add 5 mL freshly thawed trypsin (0.05% in DPBS, 5 mM EDTA).
  g. Incubate for 5min at 37°C.
    ***Note:*** Further incubation up to a total of 7min may be required. If the stroma is not starting to dissociate after this, gently titurate in the trypsin using a 5 mL serological pipette.
  h. Quench trypsin with 5 mL OP9-M media, titurate cells until stromal layer is uniformly detached and transfer to a 15 mL conical tube.
  i. Centrifuge at 300 g for 5min.
  j. Count cells and plate 70 000 – 100 000 cells into 10 mL OP9-M media into new, gelatinized 10 cm tissue culture dish (typically 1:4 split).
    **CRITICAL:** As the stroma is routinely grown to near confluence effective dissociation is essential. We recommend using trypsin from freshly thawed aliquots and meticulously washing the stroma of any residual serum-containing media twice using DPBS. Adequate mechanical dissociation is essential; scoring of the stromal matrix can assist the action of trypsin.
    ***Note:*** OP9 stroma can be passaged extensively provided morphology remains stable.
    ***Note:*** Once good quality cultures are established freeze aliquots of low passage cells at a ratio of 1:3**–**1:4 per 10cm plate in 90% qualified-FBS/10% DMSO.

10. Expand stocks of MS5 stroma.
  a. Make porcine gelatin (0.1% w/v porcine gelatin from powder in DPBS, autoclaved).
  b. Add 5 mL porcine gelatin to 75 cm^2^ flasks and incubate at 37°C for a minimum of 2 h prior to seeding cells.
  c. Make MS5-maintenance (MS5-M) media.
  d. Thaw MS5 in 10**×** volume of MS5-M media, centrifuge at 300 g for 5min and seed pellet into pre-gelatinized 75 cm^2^ flask in 12ml MS5-M media.
  e. Monitor daily and split when 80% confluent (Figure 3A).
    **CRITICAL:** Do not allow MS5 maintenance cultures to overgrow (Figure 3B).

## Key resources table

**Table:** 

REAGENT or RESOURCE	SOURCE	IDENTIFIER
**Antibodies**		

CD10-BV421	BD	Cat# 562902; RRID: AB_2737879
CD11b-APC	Biolegend	Cat# 301310; RRID: AB_314162
CD14-PECy7	Biolegend	Cat# 325618; RRID: AB_830691
CD19-PE	Biolegend	Cat# 302254; RRID: AB_2564142
CD19-APC	Biolegend	Cat# 302212; RRID: AB_314242
CD20-BV510	BD	Cat# 563067; RRID: AB_2737985
CD31-APC	Biolegend	Cat# 303116; RRID: AB_1877151
CD33-BV786	BD	Cat# 740974; RRID: AB_2740599
CD34-PECy7	Biolegend	Cat# 343516; RRID: AB_1877251
CD43 FITC	BD	Cat# 555475; RRID: AB_395867
CD45-Alexa700	BD	Cat# 560566; RRID: AB_1645452
CD45RA-FITC	ThermoFisher	Cat# MHCD45RA01; RRID: AB_10373858
CD45RA-BV711	Biolegend	Cat# 304138; RRID: AB_2563815
CD127 (IL7R)-PE	Biolegend	Cat# 351304; RRID: AB_10720185
CD335-BV421	BD	Cat# 564065; RRID: AB_2738572
IgM-BV510	BD	Cat# 563113; RRID: AB_2738010
Human Fc Block	BD	Cat# 564220; RRID: AB_2869554

**Chemicals, Peptides, and Recombinant Proteins**		

Human basic Fibroblast Growth Factor (hbFGF)	Amsbio	Cat# AMS-480-100; GenPept: P09038
Human Interleukin 3 (hIL-3)	PeProTech	Cat# 200-03; GenPept: P08700
Human FLT3 Ligand (hFLT3L)	PeProTech	Cat# 300-19; GenPept: P49771
Human Stem Cell Factor (hSCF)	PeProTech	Cat# 300-07; GenPept: P21583
Human Interleukin 7 (hIL7)	PeProTech	Cat# 200-07; GenPept: P13232
StemFit Basic04	Amsbio	Cat# SFB-504
Matrigel	Corning	Cat# 354277
Y27632 (Rock Inhibitor)	Cell guidance systems	Cat# SM02-10; CAS: 146986-50-7
Dimethyl Sulfoxide (DMSO)	Sigma-Aldrich	Cat# D2650; CAS: 67-68-5
Knock out replacement serum (KORS)	Gibco	Cat# 10828010
Gentle Cell Dissociation Reagent (GCDR)	Stem Cell Technologies	Cat# 07174
ReLeSR	Stem Cell Technologies	Cat# 05872
Qualified FBS	Gibco	Cat# 26140079
DMEM/F12	Gibco	Cat# 21331046
αMEM powder	Gibco	Cat# 12000014
Sodium Bicarbonate Solution (7.5%)	Sigma-Aldrich	Cat# S8761; CAS: 144-55-8
Distilled Water (tissue culture grade)	Gibco	Cat# 15230188
DPBS, Ca^2+^/Mg^2+^ free	Gibco	Cat# 14190169
Monothioglycerol (MTG)	Sigma-Aldrich	Cat# M6145; CAS: 96-27-5
Pencillin/Streptomycin	Gibco	Cat# 15140122
Collagenase, Type IV, powder	Gibco	Cat# 17104019
EDTA 0.5M	Sigma-Aldrich	Cat# E7889; CAS: 139-33-3
Trypsin 2.5%	Gibco	Cat# 15090046; CAS: 9002-07-7
Bovine gelatin solution (Type B, 2%)	Sigma-Aldrich	Cat# G1393; CAS: 9000-70-8
Gelatin from porcine skin (Type A)powder	Sigma-Aldrich	Cat# G1890; CAS: 9000-70-8
7-Aminoactinomycin D (7-AAD) (viability)	BD	Cat# 559925; RRID: AB_2869266
Trypan Blue (0.4%)	ThermoFisher	Cat# 15250061; CAS: 72-57-1

**Critical Commercial Assays**		

QuadroMACS Separator	Miltenyi Biotec	Cat# 130-090-976
MACS LS Columns	Miltenyi Biotec	Cat# 130-042-401
CD34 MicroBead Kit, human	Miltenyi Biotec	Cat# 130-046-702; RRID: AB_2848167

**Experimental Models: Cell Lines**		

ShiPS-MIFF1	University of Sheffield	https://hpscreg.eu/cell-line/UOSi001-A
ShiPS-MIFF3 (RRID:CVCL_1E70)	University of Sheffield	http://hpscreg.eu/cell-line/UOSi001-B
H1	WiCell	http://hpscreg.eu/cell-line/WAe001-A
OP9 stroma	ATCC	Cat# CRL-2749; RRID: CVCL_4398
MS5 stroma	DSMZ	Cat# ACC 441; RRID: CVCL_2128

**Software and Algorithms**		

Flowjo		https://www.flowjo.com
Microscope	Olympus, CKX53	N/A
Scale bar software	ImageJ	https://imagej.nih.gov/ij/

**Other**		

10-cm Tissue Culture Dish	Falcon	Cat# 353003
6-Well Cell Culture Plate	Corning	Cat# 3516
Cell Culture Flask 75cm^2^	Corning	Cat# 430641U
Disposable PES Filter Units (250 ml)	Fisherbrand	Cat# 15993297
Disposable PES Filter Units (500 ml)	Fisherbrand	Cat# 15913307

## Materials and equipment

**Table:** 

StemFit04	Final concentration	Amount
StemFit Basic04		45ml
hbFGF (Stock: 100 ng/μl)	10ng/mL	4.5μl
Penicillin/Streptomycin (optional)	1%	450μl

Store at 4°C for up to 1 week.

**Table:** 

Y27632 (ROCK Inhibitor) 1000**×** stock	Final concentration	Amount
Y27832 powder	10 mM (1000**×**)	2mg
Distilled Water (tissue culture grade)		624.4μl

Make working aliquots and store at −20°C. Use 1:1000 (final concentration 10μM).

**Table:** 

αMEM from Powder	Final concentration	Amount
αMEM Powder (10.38 mg)	1**×**	1 vial (10.38g)
7.5% Sodium Bicarbonate (NaHCO_3_) (culture grade)		29.3ml
Distilled Water (tissue culture grade)		970ml
**Total**	**n/a**	**1L**

Filter over a 0.22 μm membrane and store at 4°C for up to 1 month.

**Table:** 

Monothioglycerol (MTG) 1000**×** stock	Final concentration	Amount
MTG	100 mM	10μl
DPBS (Ca^2+^/Mg^2+^ free)	1**×**	1150μl

Make fresh.

**Table:** 

OP9-Maintenance media	Final concentration	Amount
αMEM from powder	1**×**	197.5ml
Qualified FBS	20%	50ml
Monothioglycerol (MTG) 1000**×** (100 mM)	100μM	250μl
Penicillin/Streptomycin	1%	2.5ml
**Total**	**n/a**	**250mL**

Filter over a 0.22 μm membrane and store at 4°C for up to 2 weeks.

**Table:** 

OP9-Differentiation media	Final concentration	Amount
αMEM from powder	1**×**	445ml
Qualified FBS	10%	50ml
Monothioglycerol (MTG) 1000× (100mM)	100μM	500μl
Penicillin/Streptomycin	1%	5ml
**Total**	**n/a**	**500mL**

Filter over a 0.22 μm membrane and store at 4°C for up to 2 weeks.

**Table:** 

MS5-Maintenance media	Final concentration	Amount
αMEM from powder	1**×**	445ml
Qualified FBS	10%	50ml
Penicillin/Streptomycin	1%	5ml
**Total**	**n/a**	**500mL**

Filter over a 0.22 μm membrane and store at 4°C for up to 2 weeks.

**Table:** 

Bovine gelatin	Final concentration	Amount
Bovine gelatin 2% (tissue-culture grade)	0.1%	25ml
DPBS (Ca^2+^/Mg^2+^ free)		475ml

Filter over a 0.22 μm membrane and store at 4°C for up to 6 months.

**Table:** 

Porcine gelatin	Final concentration	Amount
Porcine gelatin	0.1% w/v	0.5g
DPBS (Ca^2+^/Mg^2+^ free)		500ml

Autoclave and store at 4°C for up to 6 months.

**Table:** 

Collagenase IV 10**×** stock	Final concentration	Amount
Collagenase IV powder	10mg/mL	1g
DMEM/F12		100ml

Filter over a 0.22 μm membrane, aliquot and store at −20°C for up to 6 months.

To make 1**×** working solution dilute 10**×** in DMEM/F12. Always make fresh.

**Table:** 

Trypsin/EDTA	Final concentration	Amount
Trypsin (2.5%)	0.05%	1ml
EDTA 0.5M	5mM	500μl
DPBS (Ca^2+^/Mg^2+^ free)		48.5ml

Use fresh for OP9.

**Table:** 

MACS Buffer	Final concentration	Amount
DPBS (Ca^2+^/Mg^2+^ free)		490ml
Qualified FBS	2%	10ml
EDTA (0.5M)	2mM	2ml

Filter over a 0.22 μm membrane, store at 4°C and use within 1 month.

**Table:** 

Antibodies - FACS staining Day10	Dilution	Conjugate	Clone
CD31	1:100	APC	WM59
CD33	1:40	BV786	WM53
CD34	1:200	PECy7	581
CD43	1:40	FITC	1G10
CD45	1:80	Alexa700	HI30
CD45RA	1:40	BV711	HI100
CD127 (IL7R)	1:20	PE	A019D5
7-Aminoactinomycin D (7-AAD)	1:500		

**Table:** 

Antibodies - FACS staining Day 31	Dilution	Panel	Conjugate	Clone
CD10	1:80	B	BV421	HI10a
CD11b	1:80	Myeloid	APC	ICRF44
CD14	1:80	Myeloid	PECy7	HCD14
CD19	1:40/1:80	B/Myeloid	APC/PE	HIB19
CD20	1:80	B	BV510	2HF
CD33	1:40	B/Myeloid	BV786	WM53
CD34	1:160	B	PECy7	581
CD45	1:80	B/Myeloid	Alexa700	HI30
CD45RA	1:40	B/Myeloid	FITC	MEM-56
CD127 (IL7R)	1:20	B	PE	A019D5
CD335	1:60	Myeloid	BV421	9E2
IgM (option for CD20)	1:100	B (option)	BV510	G20-127
7-Aminoactinomycin D (7-AAD)	1:500	B/Myeloid		

Divide into two samples to stain for both mature myeloid markers and B cell progenitor markers (not all markers shown in Figure 8).

**Table:** 

Instrument	Laser wavelength	Laser power	PMTs and filter configurations
BD FACS AriaIII	Violet 405nm	30 mW	A: 780/60: B:710/50: C: 665/30: D: 610/20; E: 510/50; F: 450/40
	Blue 488nm	20 mW	A: 680/13; B: 525/50; C: 488/10
	Yellow-Green 561nm	50 mW	A: 780/60; B: 710/50; C: 670/14; D: 610/20; E: 582/15
	Red 640nm	17mW	A: 780/60; B: 730/45; C: 660/20
BD LSR Fortessa X20	UV 355nm	15 mW	A: 525/50; B: 379/28
	Violet 405nm	50 mW	A: 780/60: B:710/50: C: 670/30: D: 610/20; E: 525/50; F: 450/50
	Blue 488nm	60 mW	A: 710/50; B: 530/30; C: 488/10
	Yellow-Green 561nm	50 mW	A: 780/60; B: 710/50; C: 670/30; D: 610/20; E: 586/15
	Red 640nm	40 mW	A: 780/60; B: 730/45; C: 670/30

## Step-by-step method details

### Prepare hPSCs and overgrown plates of OP9 stroma

**Timing: 8–12 days**

Whole hPSC colonies are harvested and co-cultured on overgrown OP9 stroma for 10 days. During this time differentiation is directed towards mesodermal and later hematopoietic-competent endothelial progenitors. At day 10, CD34^+^ cells are harvested and co-cultured for a further 21 days on MS5 stroma in media supplemented with lymphomyeloid cytokines.

***Note:*** Whilst hPSCs can be readily differentiated into many other hematopoietic linages, achieving B cell differentiation is more challenging. It remains unclear to us as to whether this reflects to some unique aspect of B cell biology, or some as yet undefined aspect of the protocol. When establishing this technique we therefore stress the importance of careful validation of reagents and strict adherence to protocol.

1. Overgrow OP9 cultures.
  a. On day 4 after seeding remove 5 mL of media from OP9 plates to be used for differentiation and replace with 5 mL fresh, warm OP9-M media.
  b. The ideal timing to add hPSCs to OP9 is day 8 after seeding, but this can be extended to 10–12 days if stromal morphology remains robust. 5 mL off/on media changes should be performed every 4 days.
2. Prepare hPSCs for harvest.
  a. Passage hPSCs 3–5 days in advance of harvest for use on OP9 at such a density as to provide large undifferentiated colonies.
  b. Continue to feed with StemFit media daily or according to recommendation by manufacturer.

### Harvest hPSCs and seed onto overgrown plates of OP9 stroma

**Figure 4:**
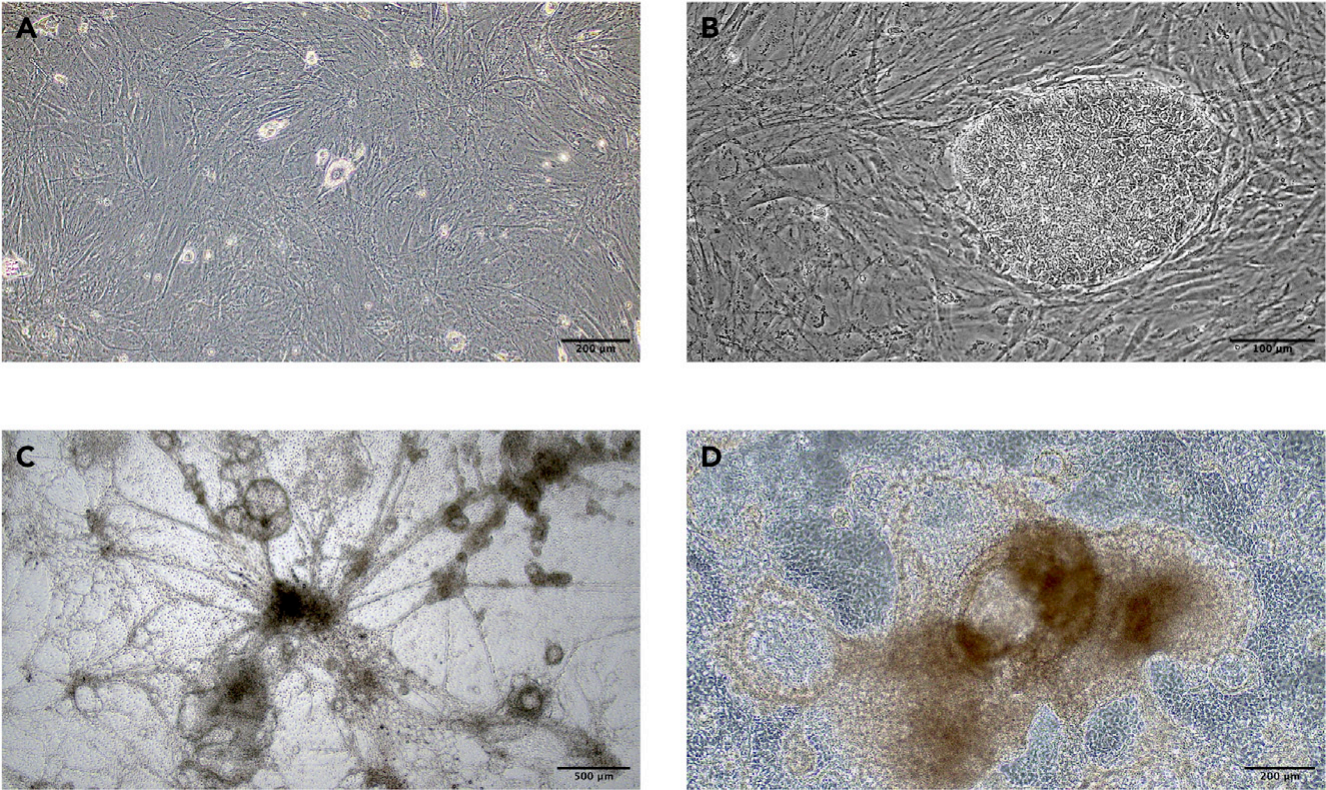
Morphological changes during hPSC-OP9 coculture (A) Morphology of overgrown OP9 stroma at 8 days post passage, ready to be seeded with hPSCs. (B) Early hPSC (MIFF3) colony growing on OP9 stroma (day 2 of co-culture). (C) hPSC (MIFF3) colony starting to differentiate on OP9 stroma (day 7 of co-culture). (D) Differentiated hPSCs (MIFF3) at day 10 of OP9 stroma co-culture at time of harvest. Microscope Olympus, CKX53. Scale bars were added using ImageJ software.

hPSCs are harvested, ideally as whole colonies or large clumps, washed and seeded onto overgrown OP9 in OP9-differentiation (OP9-D) media. The schedule of feeding of these co-cultures is critical and adhering to the timings stated here is highly recommended. During 10 days of co-culture, a series of characteristic morphological changes are observed as indicated in Figures 4A–4D.

3. Harvest hPSCs as whole colonies using collagenase.
  a. Aim to harvest between 1.5-2**×**10^6^ hPSCs (1–3 wells of a 6 well plate) per 10 cm dish of OP9.
    ***Note:*** In our experience the surface area of OP9 used rather than the number of hPSCs added is the principal determinant of CD34^+^ cell yield.
  b. Prepare fresh collagenase IV solution in DMEM/F12 basal media and warm to 37°C.
  c. Remove StemFit media and replace with 1 mL of warmed collagenase IV.
  d. Incubate at 37°C for 30–90 mins until colonies start to detach.
    ***Note:*** Some manual agitation of the plate can assist during this time, but every effort should be made to prevent break up of colonies.
  e. Once the majority of colonies are detaching, use OP9-D media to gently wash whole hPSC colonies/clumps from the well into a 50 mL conical tube using a serological pipette.
  f. The colonies are washed 3 times in 10 mL OP9-D media by gravity alone.
    **CRITICAL:** Do not pellet by centrifugation as this results in break-up of colony structure.
  g. Remove OP9-M media from overgrown OP9 plate and add 5 mL OP9-D media
  h. After the final wash, hPSC colonies are gently resuspended in 5 mL OP9-D media and transferred to the overgrown OP9 plate using a 10 mL serological pipette.
  i. Ensure that colonies are evenly distributed across the available surface area by gentle agitation.

4. Feed OP9-hPSC co-cultures.
  a. Day 1: Gently swirl plate, remove all media and hPSC cell debris and gently replace with 20 mL OP9-D media, taking care not to detach the stromal layer.
  b. Day 4: Exchange 10 mL off/on OP9-D media.
  c. Day 6: Exchange 10 mL off/on OP9-D media.
  d. Day 8: Exchange 10 mL off/on OP9-D media.

### Harvest hPSC-derived CD34^+^ cells and seed onto MS5

5. Day 9: Prepare 6 well plates of MS5 stroma.
  a. Pre-gelatinize (porcine gelatin 0.1%w/v) the required number of wells of a 6 well plate for a minimum of 2 h prior to seeding MS5.
  b. Harvest and count MS5 cells as per maintenance protocol above.
  c. Seed up to 2**×**10^5^ MS5 cells per well of a 6 well plate in MS5-M media.

***Note:*** At the time of seeding the MS5 are approaching confluency. We have not found it necessary to mitotically inactivate MS5.

**Figure 6:**
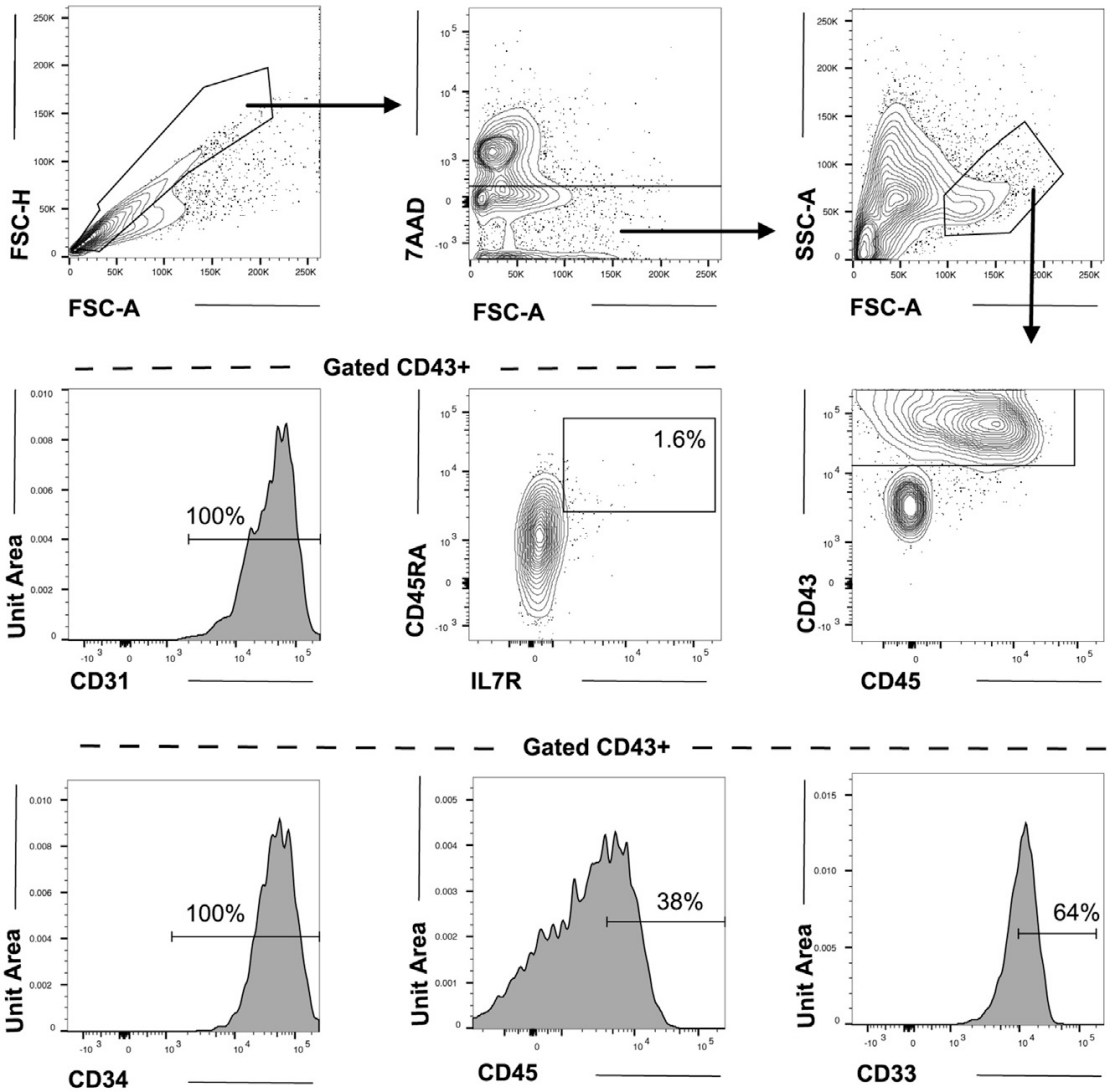
Flow cytometry analysis from day 10 of co-culture Cells were harvested after OP9 culture on day 10, enriched for CD34 and stained for surface markers. Cells were gated for singlets, viability, and scatter profile (top row) and then for CD43 expression (middle right). Of the 7AAD^-^CD43^+^ emerging hematopoietic cells, a small fraction (about 1.6% in this experiment) co-express IL7R and CD45RA (middle center). In the histograms, expression of the hematopoietic-endothelial markers CD31 and CD34 and the pan-hematopoietic (CD45) and myeloid (CD33) markers within the 7AAD^-^CD43^+^ fraction are shown. We find that about 75% of the enriched cells express the early hematopoietic marker CD43. Percentages presented are of total 7AAD^-^CD43^+^ cells.

6. Day 10: Disassociate OP9-hPSC co-culture by collagenase and trypsin digest.
  ***Note:*** By day 10 colony morphology should represent that shown in Figure 4D. Some degree of background adiposity (<20%) of the OP9 stroma is to be expected by this time.
  a. Prepare and warm fresh collagenase IV and trypsin according to description in Materials and Equipment section.
  b. Remove all media from the OP9-hPSC plate to a 50 mL conical tube on ice.
    ***Optional:*** If more than one plate is being harvested, pellet any non-adherent cells in the used media by centrifugation at 300g for 5min and discard supernatant. The disassociated stroma harvested in step 6k can be added to this pellet, thus collecting both adherent and non-adherent cells present in the co-culture.
  c. Add 7 mL warmed collagenase IV 1 mg/mL in DMEM/F12 and incubate at 37°C for 25 min. Gently swirl the collagenase to wash off residual serum-containing media.
    **CRITICAL:** The use of collagenase is the only wash-step prior to adding trypsin. For the trypsin not to be inhibited by residual FBS it is essential to remove all media carefully and swirl the collagenase to wash residual serum from the tissue culture plate.
  d. Carefully remove and discard collagenase, avoiding losing cellular material or detaching the stromal layer.
    ***Note:*** Take care not to discard cellular debris after the collagenase wash. We do not collect collagenase-containing media to reduce exposure of cells to enzyme.
  e. Add 7 mL freshly prepared 0.05% trypsin/5 mM EDTA.
  f. In order to prevent the stromal layer detaching *en-bloc* into an indigestible ball, score the stromal matrix orthogonally into approximately 1 cm^2^ sections using a 1 mL micropipette tip.
  g. Incubate at 37°C.
  h. Agitate the plate intermittently during incubation to facilitate trypsin exposure to the underside of the stromal layer.
  i. After 3–7 min use a 10 mL serological pipette to titurate the stroma, before quenching trypsin with 7 mL OP9-D media.
  j. Further titurate with a 1 mL pipette to break up clumps.
  k. Collect the cellular material into the 50 mL conical tube used in step 6b though a 40–70 μm nylon filter (pre-wetted with MACS buffer).
  l. Wash the plate with 5 mL MACS buffer and add to 50 mL conical tube through the 40–70 μm nylon filter.
  m. Wash the filter twice with 1 mL MACS buffer.
  n. Discard the filter and pellet cells by centrifugation at 300 g for 5min.

7. Magnetic separation of CD34^+^ cells.
  a. Resuspend cell pellet in 300 μL chilled MACS buffer.
  b. Incubate with 100 μL Fc receptor block for 5 min on ice.
  c. Add 100 μL anti-CD34 MACS beads and incubate at 4°C for 30 min on a rotator.
  d. Load Miltenyi LS MACS column into magnet holder, insert 30–50 μm sterile filter and place over a collection tube/reservoir.
    ***Optional:*** Flow through can be retained and analyzed by flow cytometry to estimate the relative CD34^+^ enrichment (Figure 5).
    **Figure 5:**
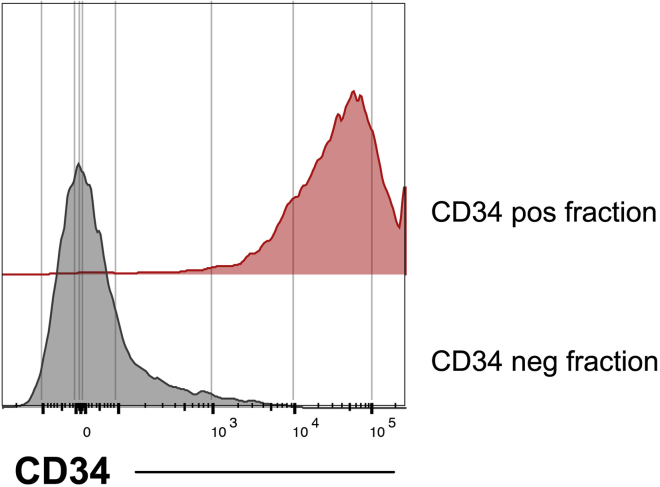
MACS enrichment of CD34^+^ cells Cells were harvested after OP9 culture on day 10 and enriched for hematopoietic progenitors using CD34 MACS beads. Cells in the CD34 positive and CD34 negative fraction were then stained for CD34 and analyzed by flow cytometry. Cells were gated for singlets, viability, and scatter profile. Histogram shows cells that express CD34 in the negative (*gray*) and positive (*red*) fraction respectively. After successful enrichment over 95% of the enriched cells are CD34^+^ and fewer than 5% CD34^+^ cells can be recovered from the CD34 depleted fraction.
  e. Prime column with 3 mL of chilled MACS buffer.
  f. After incubation wash cells with 5 mL chilled MACS buffer, centrifuge at 300 g 4°C for 5 min.
  g. Resuspend the cell pellet in 500 μL chilled MACS buffer and add to the MACS LS column through the cell strainer.
  h. Once the column stops dripping, wash with 3 mL chilled MACS buffer.
  i. When the column stops dripping, repeat 3 mL wash to a total of 3 washes.
  j. Remove the column from the magnet, discard the strainer and extract the cells by applying 5 mL chilled MACS buffer and rapid plunging the enriched cells into a fresh 15 mL conical tube. Repeat once more with 3 mL buffer to increase the yield of CD34^+^ cells.
  k. Centrifuge enriched cells at 300 g for 5 min.
  l. Resuspend the CD34-enriched pellet in 0.5 mL OP9-D media and count cells using a hemocytometer counterstained with Trypan blue. CD34^+^ cells are small, phase-bright or phase-neutral. Exclude dead (blue) or very large, phase-bright stromal cells from the cell count estimation.
    **CRITICAL:** The production of CD34^+^CD43^+^CD45^+^ hematopoietic progenitors by day 10 of co-culture is a pre-requisite for later B cell potential and therefore careful assessment of day 10 intermediate progenitors is essential when establishing this protocol. In our experience the presence of a small population of CD34^+^CD45RA^+^IL7R^+^ progenitors at day 10 is associated with robust B cell differentiation during subsequent MS5 co-culture (Figure 6). The quality of OP9 stroma and batch of the serum used are the most critical aspects of this part of the protocol.
    ***Optional:*** When establishing this protocol, we recommend staining for the presence of hematopoietic progenitors at day 10.
    ***Note:*** After this first part of the protocol harvested cells can be further cultured to generate myeloid cells as described in (Choi et al., 2011).

### Flow cytometry of hPSC-derived CD34^+^ cells

8. Take an aliquot of the CD34-enriched fraction for staining. There is no need to add more Fc blocking as this was done in step 7b.
9. Stain according to protocol below (step 15). Suggested antibodies are listed in Materials and Equipment section and typical results shown in Figure 6.

***Note:*** Trypsin/collagenase exposure may destroy surface antigens. *See troubleshooting section*.

### Lympho-myeloid differentiation of hPSC-derived CD34 cells by MS5 co-culture

10. Prepare sufficient OP9-D media with 2**×** final cytokine concentrations for the number of CD34 cells harvested.
  a. Final cytokine concentration: hIL3 10 ng/mL, hIL7 20 ng/mL, hFlt3L 50 ng/mL, hSCF 50 ng/mL.
  b. Remove MS5-M media from 6 well plate.
  c. Add 1 mL per well of 2**×** cytokine containing OP9-D media to MS5 on a 6 well plate.
11. Dilute CD34^+^ cell pellet such as to add 12.5–50**×**10^3^ CD34-enriched cells per well of MS5 on a 6 well plate in 1 mL OP9-D media. Final volume is 2 mL/well with 1**×** final cytokine concentration.
12. On day 17 of culture (day 7 on MS5) add 2 mL OP9-D media containing 1**×** cytokines: hIL7 20 ng/mL, hFlt3L 50 ng/mL, hSCF 50 ng/mL (final concentration). Final volume is 4 mL/well with 1**×** final cytokine concentration (excluding IL-3).
13. On day 24 of culture (day 14 on MS5) carefully remove up to 2 mL (accounting for evaporation – i.e., leaving 2 mL residual media) of media and replace with fresh OP9-D media supplemented with 1**×** cytokines: hIL7 20 ng/mL, hFlt3L 50 ng/mL, hSCF 50 ng/mL (final concentration). Final volume is 4 mL/well with 1**×** final cytokine concentration (excluding IL-3).

***Note:*** At this stage non-adherent hematopoietic cells should be clearly visible in the center of the well; it is therefore advisable to take media from the edge of the well. If significant numbers of non-adherent cells are removed, centrifuge the media at 300g for 5 min and reseed the pellet into the initial well in the fresh OP9-D media supplemented with cytokines as above.

***Note:*** Small numbers of CD34^+^CD19^+^ proB cells are usually detectable by day 24 of culture (after 14 days on MS5)

**Figure 7:**
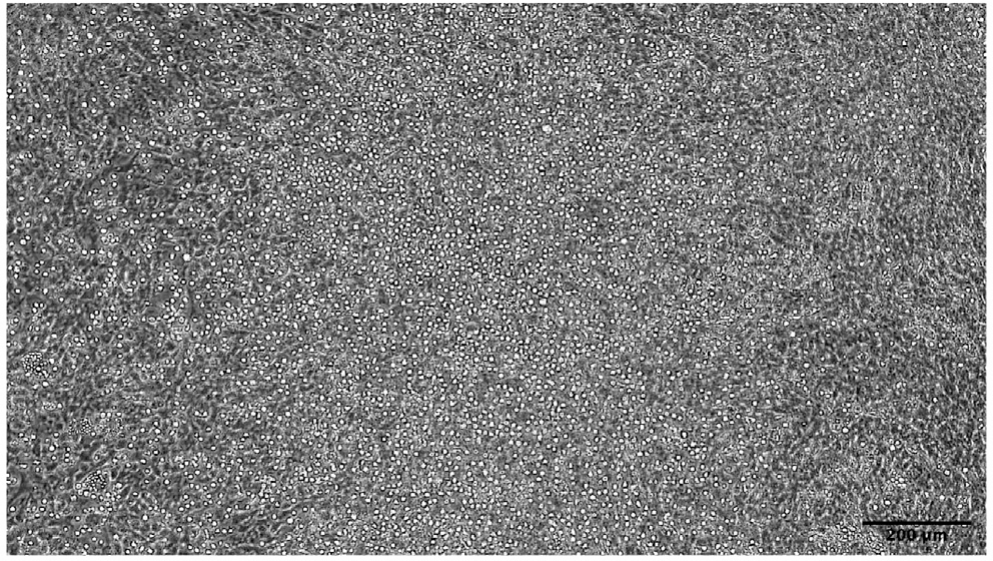
Morphology after three weeks of MS5 co-culture MS5 co-culture with hematopoietic cells one day before harvest. Small, phase bright hematopoietic cells are seen floating in the media. The MS5 stroma can be seen in the background. Microscope Olympus, CKX53. Scale bars were added using ImageJ software.

***Note:*** By day 31 the MS5 stroma usually detaches easily and enzymatic treatment with collagenase is generally unnecessary and can affect the detection of some antigens by flow cytometry.

***Note:*** By day 31 fewer primitive CD34^+^ progenitor and proB cells are present. If specific lymphoid progenitor populations are required harvesting earlier may be an advantage.

14. On day 31 (27–33) harvest cells for analysis. Small hematopoietic cells can now be seen floating in the media as per Figure 7.
  a. Use a cell scraper to mechanically detach stroma and titurate with a 1 mL micropipette into a single cell suspension.
  b. Centrifuge cells at 300 g for 5min and discard supernatant.
  c. Resuspend cell pellet in 50 μL Fc receptor block and incubate for 10 min at 4°C, aiming for a total 100 μL final staining volume. This may need to be increased if large numbers of cells are stained.
  d. Stain for surface markers according to step 15. Suggested antibodies are listed in the Materials and Equipment section.
  e. Analyze or flow sort cells for further analysis.

### Flow cytometric analysis (at day 10 to 31 of culture)

**Figure 8:**
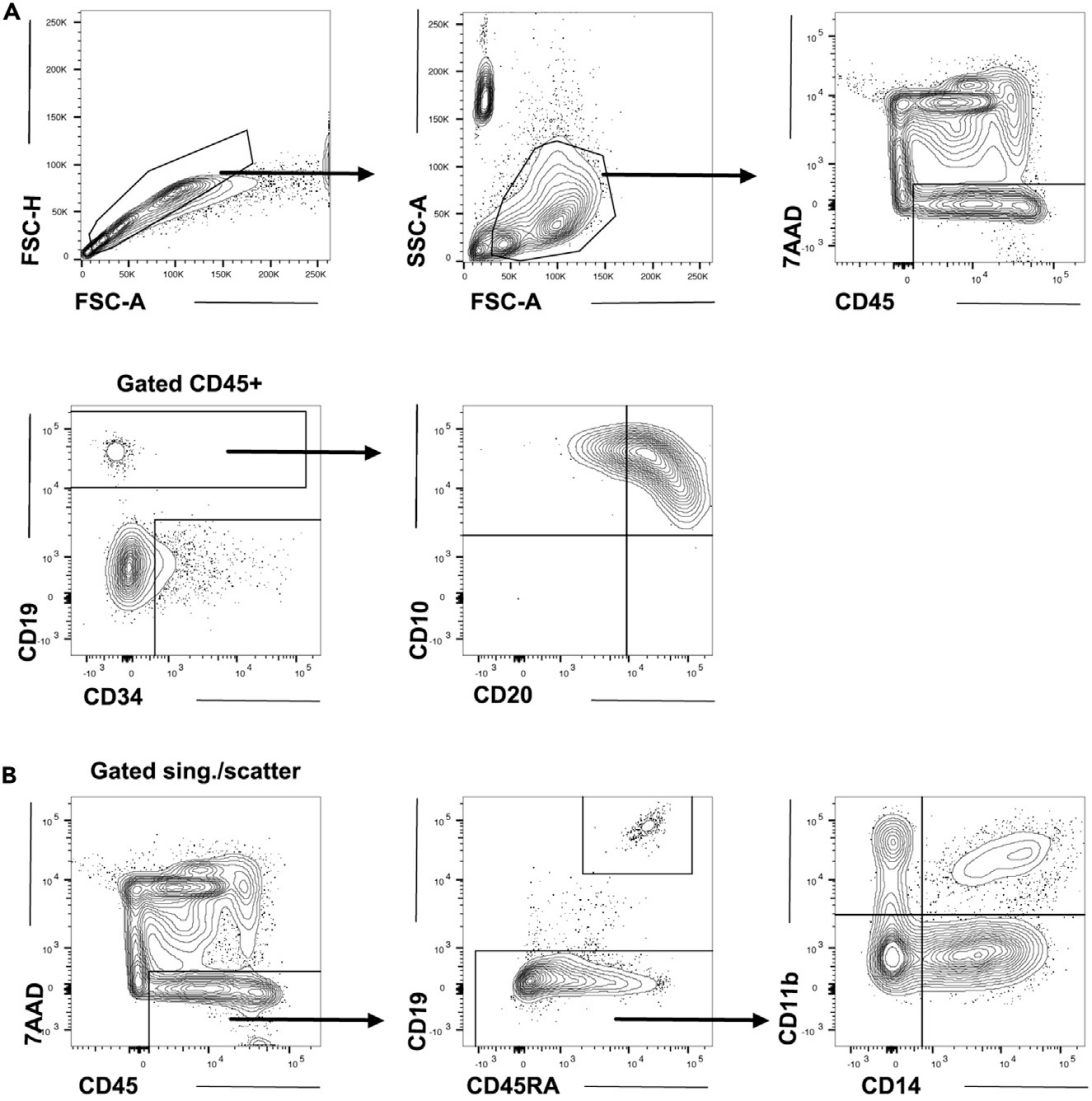
Flow cytometry analysis from approximately day 31 of co-culture Cells were harvested after 3 weeks of MS5 co-culture and stained for surface markers. (A) Cells were gated for singlets and scatter and then for viability (7AAD) and CD45 expression as indicated in the figure (top panel). Expression of CD19 and CD34 is shown within the 7AAD^-^CD45^+^ fraction (second panel, left). The CD19 positive fraction is further gated for expression of CD10 and CD20 (right). Counting beads were used in this experiment and can be seen in the scatter plot in the top middle panel. (B) Cells were gated for singlets and scatter (as in Figure 8A, plots not shown). Contour plot of viability (7AAD) and CD45 expression as shown in the figure (left). Further gating on 7AAD^-^CD45^+^ cells for B lymphoid (CD19) and, within the CD19^-^ gate, myeloid (CD11b and CD14) markers are shown to the right.

***Note:*** We recommend that you titrate the antibodies to find optimal concentration for your staining.

***Note:*** Staining volume needs to be adjusted to number of cells.

***Note:*** MS5 stroma may give a high background staining; we suggest growing and staining MS5 stroma with no hematopoietic cells as a control. We use anti-human CD45 to specifically gate on the hPSC-derived blood cells and exclude stroma that are of mouse origin.

***Note:*** Our staining suggestion was done on a BD Fortessa X20 or BD Aria III according to configuration in Materials and Equipment section. For cell sorting we recommend using 100μm nozzle to minimize mechanical stress on the cells.

15. Cells are harvested at day 10 to 31 (from day 24–31 for B cells) of culture and stained with surface markers for analysis or sorting on a flow cytometer.
  a. Make antibody staining cocktail with 2**×** concentrated antibodies in MACS buffer.
  b. Include unstained cells and fluorescence minus one (FMO) controls for all colors. A stroma only control is also recommended as the stroma may give high background.
  c. Mix equal volume of 2**×** staining cocktail and Fc receptor blocked cells.
  d. Stain in the dark on a shaker for 40 min at 4°C.
  e. Wash with 1 mL of MACS buffer.
  f. Centrifuge at 300 g for 5 min.
  g. Resuspend in MACS buffer containing 1**×** viability dye.
  h. Set up the flow cytometry with single stained beads and cells stained with only viability marker.
  i. Suggested antibody cocktails are listed in Materials and Equipment section and expected results can be seen in Figure 6 (day 10) and Figure 8 (day 31).

### Expected outcomes

At day 10 of the protocol hematopoietic progenitors can be harvested, identifiable by expression of the early hematopoietic marker CD43 and the later pan-hematopoietic marker CD45 (Carpenter et al., 2011; Vodyanik et al., 2005). In our hands, about 70%–80% of the CD34-enriched fraction express CD43 (Vodyanik et al., 2006) and of these approximately 40% also express CD45. A small fraction of cells (usually ≥0.5% of CD43^+^) express the lymphoid IL7R surface marker (Figure 6).

B cells emerge upon further co-culture on MS5; in our experience CD34^+^CD19^+^ proB cells can be detected after about 2 weeks of MS-5 culture, but kinetics may differ between different hPSC lines. After three weeks of MS5 culture (31 days total) more mature CD19^+^ preB cells emerge; these have lost expression of CD34 and homogenously express CD10 and CD20 (Figure 8). Surface IgM expression is not detected (*data not shown*).

## Limitations

The quality and provenance of the stroma used and its maintenance in appropriate conditions is critical in the specification of lymphoid-permissive hematopoietic progenitors by day 10 of culture and subsequent B cell output.

Differentiation biases in hPSC lines are likely to affect their ability to differentiate into hematopoietic progenitors and the B lineage. New hPSC lines should be evaluated alongside an hPSC line with known B lineage potential as a positive control.

Surface IgM positive naïve B cells are not generated by day 31 of culture although IgM^+^ B cells have though been reported after extended culture (French et al., 2015).

Hematopoietic progenitors derived from hPSCs have not been demonstrated to robustly engraft in immunosuppressed mice, thus limiting assessment of *in vivo* self-renewal capacity (Slukvin, 2013).

The protocol uses xenogenic stroma and serum, which makes it unsuitable as a future Good Manufacturing Practice (GMP) protocol.

## Troubleshooting

### Problem 1

Poor hPSC morphology or high cell death (before you begin - step 6).

### Potential solution

hPSC cultures are prone to spontaneous differentiation or cell death. Ensure Matrigel is used according to manufacturer’s instructions, specifically: use the dilution factor supplied with each lot, ensure that it is handled on ice until ready for use and that coated plates are not used beyond 7 days after aliquot thawing.

Different cell lines grow variably in commonly used media. We have found that H1 hES or MIFF3 hIPS cell grow robustly in mTeSR1 or StemFit (Basic04). Cell line variability can be controlled by growing a commonly used cell line (such as H1 hES or MIFF3 hIPS) in parallel. Regular media changes are essential as hbFGF is thermolabile and ongoing signaling is required for maintenance of the pluripotent stem cell state.

hPSC colonies should not be allowed to overgrow and passaging frequency will need to be optimized depending on culture conditions to achieve morphology seen in Figure 1A. Smaller areas of differentiation are often selected against during passage, but if problematic can be removed either manually or with ReLeSr reagent during passage.

### Problem 2

Poor OP9 morphology (before you begin - step 9).

### Potential solution

Maintaining stable OP9 cultures with a morphological profile similar to that shown in Figure 2 is essential for generating hematopoietic progenitors. The OP9 we use for hPSC differentiation are handled differently to those we use for standard hematopoietic differentiation assays and stored as separate sub-lines. We therefore recommend passaging new OP9s using the conditions described above until stable morphology is achieved before freezing down working stocks. Specific attention should be paid to: i) choice of plastic of cell culture dishes; ii) adequate gelatinization of cell culture dishes; iii) use of qualified, batch-tested serum; iv) use of basal αMEM media made from powder; v) seeding density; vi) passaging completely using fresh aliquots of trypsin at 80% confluence.

### Problem 3

There are few/no CD34^+^CD43^+^ hematopoietic cells at day 10 of OP9 co-culture (step 8).

### Potential solution

The problem is most likely the quality of the OP9 stroma. Ensure you are using the correct type of tissue culture plastic and that this is well gelatinized. The stroma is sensitive to inter-batch variation in qualified FBS and the basal αMEM media should be made from powder. Do not allow OP9 to overgrow during maintenance phase and ensure complete trypsinization at passage. Higher passage OP9 can become exhausted. Good quality OP9 should appear as in Figures 2A and 2B in the maintenance phase and Figure 4A before start of the co-culture with hPSCs.

Another reason can be variability in the differentiation potential of the hPSC line used. Include a control cell line that is known to produce blood cells such as H1 hES, MIFF1 or MIFF3 iPS cell lines. Ensure hPSC cultures are stable and free from significant baseline differentiation.

### Problem 4

No B cells (CD19^+^) at day 31 of co-culture (step 14).

### Potential solution

Most likely there is a problem with either the OP9 stroma, MS5 stroma or the qualified serum. If you see expression of CD43 and CD45 and a small population of IL7R positive cells at day 10 then the OP9 stroma is probably satisfactory. We then suggest you test your MS5 culture system using cord blood-derived progenitor cells in parallel to confirm B lineage output. Lineage negative CD34^+^CD38^-^CD45RA^+^CD10^+^ cord blood cells should, for instance, give robust B cell output in bulk MS5 co-culture (Doulatov et al., 2010).

There could also be a general differentiation issue with the hPSC cell line used. We therefore recommend establishing the protocol with a well maintained hPSC line with known B lineage potential such as H1 hES, or MIFF1 / MIFF3 iPS cell lines.

### Problem 5

Flow cytometric analysis shows lack of expression of a specific surface antigen (step 9 and 15).

### Potential solution

Some surface antigens are sensitive to collagenase/trypsin treatment. To test if this is the reason, cells known to express the surface antigen (e.g., control cord blood) can be stained with and without prior exposure to collagenase and/or trypsin treatment and analyzed. If the surface antigen is sensitive to the enzymatic degradation, we suggest reducing exposure to the enzyme or trying a different clone of antibody, if available.

## Resource availability

### Lead contact

Further information and requests for resources and reagents should be directed to and will be fulfilled by the lead contact, Simon Richardson (ser32@cam.ac.uk).

### Materials availability

This study did not generate new unique reagents.

### Data and code availability

No new code was established in this study.
